# Ecological dynamics of field *Aedes albopictus* populations under *Wolbachia*-mediated suppression

**DOI:** 10.1186/s40249-025-01367-9

**Published:** 2025-09-24

**Authors:** Yongjun Li, Jun-Tao Gong, Yongkang Liang, Linchao Hu, Yingyang Wei, Renxian Gan, Xiaohua Wang, Jianshe Yu, Moxun Tang, Ary A. Hoffmann, Bo Zheng, Zhiyong Xi

**Affiliations:** 1 Department of Pathogen Biology, School of Medicine, Jinan University, Guangzhou, 510632 China; 2 Guangzhou Wolbaki Biotech Co., Ltd, Guangzhou, 510700 China; 3 School of Mathematics and Information Science, Center for Applied Mathematics, Guangzhou University, Guangzhou, 510006 China; 4 Department of Mathematics, Michigan State University, East Lansing, MI 48824 USA; 5 Bio21 Institute, School of BioSciences, University of Melbourne, Parkville, VIC 3010 Australia; 6 Department of Microbiology, Genetics, Immunology, Michigan State University, East Lansing, MI 48824 USA

**Keywords:** *Wolbachia*, Standalone incompatible insect technique, *Aedes albopictus*, Ecological dynamics, Population suppression, Population replacement

## Abstract

**Background:**

The incompatible insect technique (IIT), based on *Wolbachia*-induced conditional sterility, has proven highly effective in suppressing mosquito populations for dengue control. However, concerns that accidental release of infected females could drive population replacement have prompted integration of IIT with irradiation or advanced sex-separation technologies. Moreover, the broader ecological consequences of IIT-based suppression remain insufficiently understood. Here, we investigated whether standalone IIT, leveraging *Wolbachia*-associated fitness costs under real-world conditions, can effectively suppress *Aedes albopictus* populations without causing replacement, while also addressing key ecological concerns related to IIT-based mosquito population suppression.

**Methods:**

We conducted field trials on Shazai Island, Nansha District, Guangzhou, China, releasing approximately 16,000 *Wolbachia* *w*Pip-transinfected *A. albopictus* HC males per hectare per week from 2018 to 2019, following three years of combined IIT and sterile insect technique (SIT) application. Population suppression was monitored, with *w*Pip infection frequency assessed to evaluate population replacement risks. Two-dimensional system of ordinary differential equations incorporating *Wolbachia*-induced fitness costs was established to predict population dynamics. Additionally, we assessed female mating preferences after three years of suppression and the impact on non-target *Culex quinquefasciatus* populations.

**Results:**

We offer both empirical evidence and a mathematical model, demonstrating that the fitness costs associated with a *Wolbachia* triple-strain infection in *A. albopictus*, especially in adverse field conditions, empower a standalone IIT to effectively suppress mosquito populations without causing population replacement. Remarkably, reducing the previous release numbers to just 20% sustained a similar suppression level. We found no evidence of changes in female mating preferences after a three-year field suppression. The suppression of *A. albopictus* does not impact the population of the coexisting nontarget species *C. quinquefasciatus*. After stopping releases, the population rebounded partially in Year 1 and appeared to fully recover in Year 2, with the rate of this recovery likely influenced by mosquito immigration associated with population flow.

**Conclusions:**

Our study demonstrates the robustness, cost-effectiveness, scalability, and ecological safety of IIT as a tool for controlling mosquito-borne diseases. These findings support the implementation of field-applicable, low-dose IIT for sustainable dengue control.

**Graphical Abstract:**

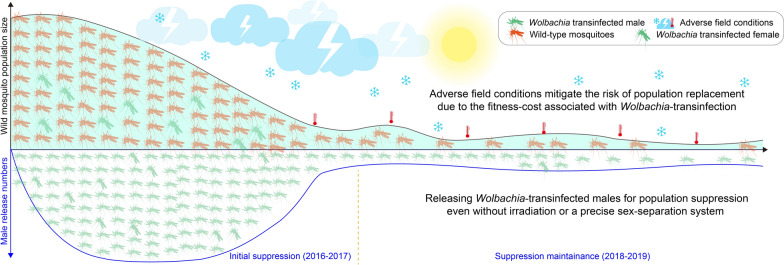

**Supplementary Information:**

The online version contains supplementary material available at 10.1186/s40249-025-01367-9.

## Background

Anthropophilic mosquitoes and the diseases they transmit impose immense burdens on human health and the global economy annually. Despite extensive efforts, the upward trajectory of mosquito-borne diseases remains unbroken [1]. Highly effective novel technologies, either as alternatives or complements to conventional mosquito control methods, are urgently required to disrupt the disease transmission. In recent years, the strategy of controlling mosquito populations through the inundative release of sterile males to induce non-reproductive matings has emerged as a promising component of integrated mosquito management programs aimed at combating these disease vectors [2]. Various approaches exist for generating sterile males for population suppression, typically involving irradiation [3], genetic modification [4] and *Wolbachia* transinfection [5]. Among them, the *Wolbachia*-based approach offers unique advantages, as infected male mosquitoes generally induce complete sterility with minimal fitness costs [6, 7], thus promising better field performance. Additionally, infected females exhibit resistance to arboviruses [8–11], reducing biosafety concerns regarding implementation. *Wolbachia* is the most prevalent endosymbiotic bacterium in nature, infecting approximately 50% of all arthropod species [12–14] but is incapable of infecting vertebrates [15, 16]. In recent years, *Wolbachia*-based mosquito population suppression, known as the incompatible insect technique (IIT), has been implemented in numerous countries, leading to a substantial reduction in *Aedes* mosquito populations, significantly contributing to the mitigation of mosquito-borne diseases by decreasing mosquito biting [17–21].

IIT faces the risk of population replacement due to the reproductive advantage conferred by *Wolbachia*-infected females over uninfected ones through cytoplasmic incompatibility (CI), where early embryonic death occurs when an infected male mates with either uninfected females or females infected with a different *Wolbachia* strain [22, 23]. During the implementation of IIT, the unintentional release of fertile *Wolbachia*-infected female mosquitoes, typically accompanied by a large number of *Wolbachia* transinfected males, into the field has the potential to cause population replacement. This occurs when *Wolbachia* invades the targeted mosquito population, resulting in the entire population becoming infected [24, 25]. Consequently, the ongoing release of males will no longer lead to female sterility, as the field population becomes compatible with the released males. The predominant method for mosquito sex-separation relies on the gender-associated pupal size difference. Due to the lack of a clear boundary between the sizes of male and female pupae, this method is not precise [18, 21, 26], inevitably leading to the unintentional release of females into the field. To prevent this risk, it is believed to be necessary to entirely eliminate any fertile females that might be mixed in the males. The combination of IIT with sterile insect technique (SIT), known as combined IIT-SIT, aims to sterilize the residual females. Given that female pupae are more sensitive to irradiation than male pupae, combined IIT-SIT requires a relatively low radiation dose [21, 27], thereby reducing the adverse effects on male fitness [28, 29]. Additionally, evidence suggests that irradiated *Wolbachia*-transinfected female mosquitoes maintained low vectorial capacity [30]. To enhance the accuracy of sex sorting, Verily Life Sciences has developed an artificial intelligence (AI)-based technology with a female contamination rate of approximately 1 in 900 million [19]. While these technologies effectively mitigate the risk of population replacement, their high cost limits their widespread adoption.

However, the concern over *Wolbachia*-induced population replacement in IIT may be overstated. In practical applications of pathogen-suppressing *Wolbachia* to invade wild mosquito populations, regular female releases are often necessary initially to help establish *Wolbachia* infections to a threshold frequency [31, 32], due to *Wolbachia*-induced fitness costs [33, 34] and instability of transinfection under extreme environmental conditions like heatwaves and drought [35, 36]. Furthermore, the mere establishment of a *Wolbachia*-infected mosquito population in the field does not guarantee its perpetual existence. There have been instances where established *Wolbachia*-infected populations collapsed after experiencing hot and dry seasons [36] even when equilibrium frequencies suggested persistence [31]. Recent field trials in Changsha, a Chinese inland city with long and cold winters, demonstrated that standalone IIT using the *Wolbachia* *w*Pip transinfected *A. albopictus* HC males, with an average female contamination rate around 0.13%, can achieve over 90% population suppression without leading to population replacement [37]. These findings suggest that *Wolbachia*-mediated mosquito population replacement may not occur as readily as previously hypothesized [25, 32]. Instead, the successful establishment of *Wolbachia* in wild mosquito populations appears to hinge on the degree of CI offsetting the *Wolbachia*-induced fitness cost. Achieving this outcome requires a release strategy with purposeful design and execution. Accordingly, the risk of population replacement in IIT is conditional and should be evaluated under real-world conditions, taking into account the impact of adverse climate factors on the fitness of *Wolbachia* transinfected mosquitoes, rather than relying solely on stable laboratory conditions.

The successful field demonstration of population suppression through IIT also raises new questions concerning the deployment of this technology for broader disease control. These issues pertain to enhancing cost-effectiveness by reducing the required number of released mosquitoes for maintaining suppression, assessing the potential development of resistance in field females to mate with incompatible males after prolonged suppression, examining the responses of the coexisting non-target mosquito species to population suppression, and gauging the rate of population rebound after releases stop. Addressing these questions necessitates a full evaluation and the refinement of this technology through field trials.

In this study, we’ve leveraged our extensive experience in mass-producing *Wolbachia*-transinfected *A. albopictus* HC mosquitoes since 2014, along with field trials involving HC male releases dating back to 2015. We have shown that in the context of mosquito control via IIT, the occurrence of population replacement is contingent upon specific conditions, closely tied to the extent of *Wolbachia*-associated fitness costs in adverse field conditions and the level of population suppression. We have demonstrated that the standalone IIT with low-dose (approximately 20% of previous release numbers) release of *A. albopictus* HC males can effectively maintain the suppression, even in the presence of imprecise sorting of males and females. After releases were stopped, the population rebounded to approximately half the size of the control site in Year 1 and a size similar to the control site in Year 2. We have also provided evidence on the impact of 3 year suppression on *A. albopictus* female mating preferences and the population of the non-target mosquito species *Culex quinquefasciatus*. These results point to the robustness and cost-effectiveness of IIT-based mosquito population suppression.

## Methods

### Description of study areas

The general characterization of the study areas was described previously [21]. The Shazai and its control site, Xiaohu Island, are in Nansha District, Guangzhou, China. The Dadaosha Island and its control site, Guanlong Island, are in Panyu District, Guangzhou, China. During this study, the residential environment had been changed in Shazai Island (release site 1) and Xiaohu Island (control site 1). Specifically, in the northern part of Shazai Island (previously described as zone 20–22), all the residential buildings were torn down in early 2018. Therefore, these areas were excluded from this study. In early 2019, residents of Xiaohu Island started to move out gradually due to the impact of the nearby chemical plant explosion in 2018. In June 2019, residential buildings on Xiaohu Island began to be torn down, and several main roads were blocked in September, so we had to stop mosquito surveillance in early October. The previous release site 2 (Dadaosha Island) and its control sites (Dadaosha Island, control site 2a; Guanlong Island, control site 2b) had minor residential environment changes during this study. But one small area, between a road and zone 8 in release site 2, had changed significantly. The western part of this area used to have poor sanitation, and had a large number of mosquito breeding sites; the eastern part of this area was a buffer zone (by releasing HC males) during mosquito release, to prevent the mosquitoes from immigrating from its western part. But at the end of 2017, the western part was transformed into an outdoor activity place for villagers, thereby eliminating those mosquito breeding sites. Besides, the intensity of local mosquito control activities increased substantially in 2019, as those villages committed to building “beautiful countryside”, which was promoted by the municipal government.

### Mosquito colonies

The wild-type *A. albopictus* Guangzhou Line was newly established by collecting about 200 *A. albopictus* larvae from each of 10 scattered locations in Guangzhou City and then pooling them together and rearing them into adults. The first generation and descendants of these mosquitoes were named as GUA. The *A. albopictus* HC line was previously described and mass-reared at Guangzhou Wolbaki Biotech Co., Ltd (hereafter referred to as Wolbaki), which is infected by *w*Pip, *w*AlbA, and *w*AlbB [21]. To better represent its quality in the field, different from the previous studies [38], we outcrossed the HC line with the above GUA, which was reared in the laboratory for $< 2$ generations after field collection, for four generations, and then self-crossed for 1 generation, designated as oHC. The first generation of the oHC line was used for further experiments.

Two wild-type *A. albopictus* lines used for the female mating preference experiment (see below) were derived either from Shazai Island or Xiaohu Island and herein designated as szGUA and xhGUA, respectively. To establish these colonies, at the end of 2017 (3 years after mosquito releases), pupae or larvae were collected from 30 to 40 scattered ovitraps (Zhikehongrun Environmental Protection Technology Co., Ltd, Zhengzhou, China) and then pooled together, and reared to adults (G0). Offspring of G0 (G1) were used in the experiment, and they were reared under standard conditions and standard protocol as described before [38]. Briefly, larvae and adults were maintained in a standard mosquito-rearing room at 27 ± 2 $^{\circ }$C and 75 ± 5% relative humidity, with a 12∶12-hour light: dark photoperiod. Eggs were hatched with hatching solution [1% (w/v) yeast powder in dHO], and the newly hatched larvae were reared in trays (38 cm $\times$ 25 cm $\times$ 5 cm) at a density of 300 larvae per 1.0 L tap water per tray. Dry bovine liver powder (weighted and suspended in 5–10 ml of water before use) was supplied to the 1 st and 2nd instar larvae at 0.5 mg per larva per day and 1.0 mg per larva per day for the 3rd and 4th instar larvae. Adult mosquitoes were provided with fresh 10% sucrose solution and allowed to feed on a mouse to lay eggs.

### Mosquito survivability under stressed conditions

To study the survival rate of larvae under malnutrition, 100 newly hatched 1 st instar larvae of GUA, HC, and oHC were placed into plastic boxes (17 cm $\times$ 11.8 cm $\times$ 5.5 cm) with 500 ml of water. A bovine liver powder solution (prepared by suspending 5 g of dry bovine Liver powder in 100 ml of tap water) was used as larval food. To create a malnutrition condition, 200 ml of Liver powder solution was provided every 3 days (on average, 0.33 mg per larva per day), until all larvae developed into adults or died. There were three replicates for each mosquito line, and the food supplement in the control group was as described above. To study the larvae survival under high temperatures, first instar larvae of GUA, HC, and oHC (50 larvae in plastic boxes, 17 cm $\times$11.8 cm $\times$ 5.5 cm, with 300 ml tap water) were reared in a climate chamber with cyclical temperatures: 39 $^{\circ }$C during the 12-h Light period and 29 $^ {\circ }$C during the 12-h dark period. Larvae were reared under such conditions for 5 days before being transferred to standard rearing conditions as described above. Then count the number of larvae that successfully developed into pupae until all larvae died or pupated. To study female adult longevity under stressed conditions, 30 newly emerged female adults of GUA, HC, and oHC were placed in cages (30 cm $\times$ 30 cm $\times$ 30 cm), and a bottle of tap water without sugar was provided. Dead adults were counted every 24 h until all mosquitoes died.

### Diapause induction

The diapause induction procedure was as previously reported with modifications [39]. Briefly, for diapause induction, mosquitoes at all developmental stages were kept at 21 $^{\circ }$C with a 6∶18 light: dark cycle, and mosquitoes reared under 21 $^{\circ }$C with an 18∶6 light: dark cycle served as the control group. Approximately 300 first-instar larvae of GUA, HC, or oHC were reared in a plastic tray (as described above), and dry bovine liver powder was provided as larvae food on demand. Pupae were collected and moved to a cage (30 cm $\times$ 30 cm $\times$ 30 cm) provided with 10% sucrose solution. Two weeks later, blood-fed those mosquitoes with mice, and then 3 days later, an oviposition cup was provided to collect eggs. These eggs were kept wet under the same conditions for 10 days to allow for embryo maturation. To judge the egg hatch rate and diapause rate, the eggs were hatched under their corresponding conditions continuously for 24 h, then the egg papers were dried for 1 week, and then the eggs were hatched for another 24 h. Egg hatch rate was calculated by dividing the de-capped eggs by the total eggs immersed in water. Since during winter, the eggs of *A. albopictus* that can be hatched are biologically dead, we focused on the unhatched eggs, which could be either in a diapause state or undeveloped. To differentiate between these two types of unhatched eggs, a bleaching process was performed using a 5% sodium hypochlorite solution (Zhongding Biotech, Tianjin, China). The determination of dead or diapause status was carried out according to a previously described method [40]. Three replicates were performed, and each replicate involved randomly collecting and bleaching around 30 eggs in each group. The rate of unhatched eggs was calculated by dividing the number of unhatched eggs by the total number of eggs. The diapause rate was calculated by the number of diapause eggs divided by the total number of unhatched eggs. The proportion of diapause eggs was calculated by multiplying the rate of unhatched eggs by the diapause rate.

### Mosquito mass production, transportation, and field release

Male mosquito pupae of HC line were mass-produced in Wolbaki following the standard protocol as described previously [21]. Then about 40,000 male pupae were placed in an eclosion cage (120 cm $\times$ 30 cm $\times$ 30 cm) with cotton balls soaked with 10% sucrose water placed on the top of it. After pupae emergence, the containers for pupae were taken out, and then those cages were moved to a chilling room with a temperature of $8 \pm 2$ $^\circ$C, to knock down the adult mosquitoes so that they can be packaged. About 40,000 mosquitoes were packaged in one plastic box with a cap (27 cm $\times$ 19 cm $\times$ 3 cm). Packaged mosquitoes were then stored in a portable refrigerator (Alpicool, Foshan, China, 75 L in volume) at a temperature of $10 \pm 2$ $^\circ$C for transportation, and released immediately after they arrived at the field site (Shazai Island). During the whole process, mosquitoes were kept at low temperatures for about 2.5 h. The handling process has been proven to have a minor effect on males’ fitness [41]. To release the chilling-paralyzed mosquitoes, packages were taken out of the refrigerator, caps were removed, and mosquitoes gradually woke up by sensing the rising temperature and flew away. Mosquitoes were released while walking, but preferably released near vegetation. In 2018 and 2019, HC males were released twice a week (on Wednesday and Saturday) in Shazai Island. On average, about 400,000 and 370,000 males were released weekly in 2018 and 2019, respectively. During this study, no mosquito was released on Dadaosha Island, and at the end of 2019, release activity was stopped on Shazai Island.

### Monitoring mosquito populations in the field

The population of *A. albopictus*, as well as *C. quinquefasciatus*, was monitored from March to October between 2016 and 2019 by BG-Sentinel Traps (Biogents, Regensburg, Germany) in Shazai and Xiaohu Island, but in Dadaosha Island and its control sites, the mosquito population was monitored by ovitraps only. The operation procedures of BG traps and ovitraps were the same as described before [21]. Briefly, BG traps continuously run for 24 h every Tuesday. The captured adults, both *Aedes* and *Culex*, were sent back to the lab for species identification and quantification. Ovitraps were placed in the field for a week (usually from Monday to Monday), and those with eggs present were collected and taken back to the lab to count the hatched larvae. In 2018, in the release sites and control sites, the distributions and locations of BG traps and ovitraps were the same as previously described (2 BG traps and 5 ovitraps in each zone). But in 2019, in order to reduce manpower, the distribution density of BG traps and ovitraps was decreased. Specifically, 20 BG traps were distributed in Shazai Island (generally 1 BG trap in each zone) and 15 BG traps in Xiaohu Island; 20 ovitraps were placed on Dadaosha Island (2 to 3 ovitraps in each zone) and its control sites.

### Release ratio calculation

To reduce the cost, the release ratio (HC male to wild-type male) was calculated by the numbers of mosquitoes captured by BG traps in both the control site and release site, rather than PCR assay used from 2018 to 2019. Firstly, calculate the ratio *r* of male to female according to the numbers of male and female *A. albopictus* in the BG traps in the control site. Let *n* and *m* denote the numbers of males and females in the release site, respectively. Then the release ratio *R* is given by $R=\frac{n}{r\times m}-1$. If no female was caught in either the release site or the control site, the denominator becomes 0. In such cases, this particular data was disregarded.

### Female mating preference assay

*A. albopictus* were collected from the field in Shazai Island 3 years after release and in Xiaohu Island, which experienced no release. Virgin female adults from the first-generation (F1) offspring of the field-collected mosquitoes were used in the dual-choice experiment to mate with GUA males (F1) from Shazai and HC males, following a protocol as previously described with modifications [42]. Specifically, 2–3 day-old GUA and HC male mosquitoes were first transferred into the 30 cm $\times$ 30 cm $\times$ 30 cm cage in three different ratios, 1∶1, 1∶5 and 1∶10, and on the 2nd day, females were introduced into the cages (Table S1). All cages maintained a similar mosquito density, with 350–360 mosquitoes per cage, to avoid a density-dependent effect on mating. Mosquitoes were allowed to mate for 24 h, then male mosquitoes were removed by an aspirator. Three days later, females were blood-fed with mice. Mosquitoes that took blood were collected and reared individually in 50 ml conical centrifuge tubes supplied with wet filter paper at the bottom for egg-laying. Ten days later, those eggs were hatched by adding hatching solution into each tube and hatched for 48 h. As validated in the control crosses (Table S1), GUA females mated with HC males do not produce viable progeny, while those mated with GUA males produce mostly viable eggs. Therefore, we used egg viability as a proxy to determine whether GUA females mated with GUA or HC males. The female mating choice indices (FMCI) towards HC males or GUA males were calculated as: FMCI to HC males = $(N / R) / (Y + N / R)$; FMCI to GUA males = $Y / (Y + N / R)$, in which *N* is the number of GUA female individuals producing unviable eggs, *Y* is the number of GUA female individuals producing viable eggs, and *R* is the release ratio of GUA male vs HC male.

Based on the hypothesis that females with a preference for mating might be more resistant (but not necessarily reject) mating with unqualified males, it is expected that males would take longer to successfully engage females in stable mating pairs. We performed another experiment to assess the strength of female mating preference. We put 5 virgin males (1–2 days old, of the same line) in a 30 cm $\times$ 30 cm $\times$ 30 cm cage and supplied 10% sucrose water solution. Then, 24 h later, one virgin female mosquito (1–2 days old) was released into the cage, and immediately started timing to record how much time a male would take to seize the female to form a stable mating pair (the mating pair will rest on the cage surface). This experiment was repeated 10 times for each mating group, and here we performed three mating groups: szGUA female $\times$ HC male, szGUA female $\times$ xhGUA male, and xhGUA female $\times$ HC male.

### Monitoring *w*Pip infection frequency in the field

During this field trial, *w*Pip infection frequencies were monitored in larvae collected by ovitraps or female adults collected by the human landing method. From 2018 to 2020, about 95 ovitraps were distributed on Shazai Island, and the positions of those ovitraps were the same as previously located [21]. Egg-positive ovitraps were sent back to the lab for *w*Pip detection weekly. Because the release activity was stopped at the end of 2017 on Dadaosha Island, so we did not work on the *w*Pip detection of the larvae in the ovitraps. Starting in July 2019, larva samples from ovitraps on Dadaosha Island were collected and analyzed via PCR assays to determine whether population replacement had occurred. Furthermore, female adults were collected on Shazai Island, in 3 independent human-landing catch activities in 2020, to monitor the *w*Pip infection. Selecting locations for conducting human landings was the same as described previously [21]. In brief, we selected collection sites in close proximity to houses, often in shaded areas, where mosquitoes were readily accessible. Mosquito collection was conducted between 9:00 and 11:00 or between 16:00 and 18:00 using a mosquito aspirator. At each location, a staff remained present for 15–30 min to capture female mosquitoes. The captured females were taken to the lab for PCR assay. The PCR assay of *w*Pip infection in larvae and adults was the same as described previously [21].

### Statistical analysis

All data were statistically analyzed by SPSS 13.0 (IBM, Chicago, USA) and GraphPad Prism 6.0 software (GraphPad Software, San Diego, USA). The significance of mosquito population size between release sites and control sites, and the release ratio between different years, was determined using the two-tailed Mann Whitney U test. Indices of female mating choice to different mosquito males were compared by two-tailed *t*-test. Mating pair formation time between groups, the mosquito population size across different years, and the mosquito population recovery level across different zones were compared by one-way ANOVA. The significance of larval survival rate, egg hatch rate, diapause rate, and proportion of diapause eggs of different mosquito lines was analyzed by one-way ANOVA followed by Tukey’s multiple comparisons test. The log-rank (Mantel-Cox) test was used to compare mosquito longevity. Pearson correlation test was used to compare the *C. quinquefasciatus* population dynamics. The temporal distribution of *w*Pip-positive larvae pools was compared by Fisher’s exact test. The difference in yearly egg suppression efficiency in 2017 and 2018 across different zones was analyzed by a paired *t*-test. When the *P*-value is less than or equal to 0.05, we consider that there was a significant difference between the compared data.

## Results

### *Wolbachia*-associated fitness cost under the real-world field conditions

**Fig. 1:**
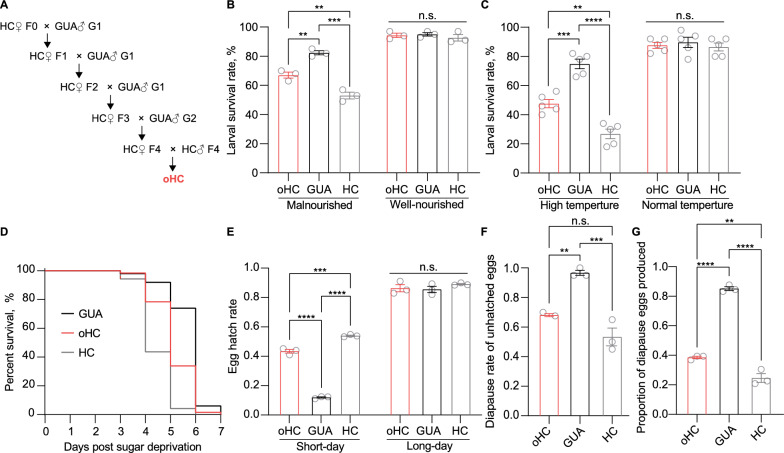
The survivability of oHC, HC and wild-type GUA in adverse field conditions. **A** The scheme of the generation of oHC line to homogenize the genetic background of HC and GUA. The larval survival rate of oHC and HC was significantly lower under **B** malnutrition ($$n = 3$$) and **C** high cyclical temperatures ($$n = 5$$). **D** Female adults of oHC and HC exhibited significantly reduced longevity under stressed conditions ($$n = 3$$). **E** After diapause induction, oHC and HC females produced a significantly higher proportion of hatched eggs ($$n = 3$$). **F** The diapause rate in unhatched eggs of oHC and HC was significantly lower than that of GUA ($$n = 3$$). **G** The proportion of diapause eggs laid by oHC and HC females was significantly lower than that of GUA ($$n = 3$$). Data in **B**, **C**, **E**, **F**, and **G** are presented as mean ± SEM. GUA indicates the wild-type *A. albopictus* lines, HC indicated that *Wolbachia*-*w*Pip transinfected *A. albopictus* line, oHC indicates the out-crossed HC line. The significance of larval survival rate, egg hatch rate, diapause rate of unhatched eggs, and proportion of diapause eggs produced across different mosquito lines was analyzed by using one-way ANOVA followed by Tukey’s multiple comparisons test. $$* P<0.05; ** P<0.01; *** P<0.001; **** P<0.0001$$. Female longevity was compared by the Log-rank test

Under favorable laboratory conditions, the long-term mass-reared HC line demonstrated robust fitness compared to their wild-type counterparts [38]. To better represent their quality in the field, we assessed the fitness of the HC line in parallel with the outcrossed HC (oHC), which was derived from four consecutive generations of crosses between HC and wild-type GUA mosquitoes, recently collected from the field, to mitigate the influence of laboratory adaptation on the evaluated traits (Fig. 1A). To simulate the adverse environmental conditions encountered by mosquitoes after release into the field in Guangzhou, we first compared the larval survivorship of oHc, HC, and wild GUA Lines under restricted food access and diurnal temperature fluctuations ranging from 29 to 39 $^\circ$C. We observed that, under normal nutritional conditions, there was no significant difference in the larval survival rate among all mosquito lines [Fig. 1B, $F(2, 6) = 0.5270, P = 0.62$]. However, under malnutrition conditions, both oHC and HC exhibited significantly lower larval survival rates compared to GUA (GUA: 82.3 ± 1.19%; oHC: 67.0 ± 1.70%; HC: 53.0 ± 1.89%; oHC versus GUA: $P = 0.0037$; HC versus GUA: $P = 0.0001$), with oHC showing significantly better survival than HC ($P = 0.0059$) (Fig. 1B). Similarly, the larval survival rates of all mosquito lines showed no difference under normal rearing temperatures (Fig. 1C, $F(2, 12) = 0.3311, P = 0.72$). However, under high cyclical temperatures, both oHC and HC displayed significantly reduced survival rates compared to GUA (GUA: 74.80 ± 2.92%; oHC: 47.60 ± 2.55%; HC: 26.80 ± 2.92%; oHC versus GUA: $P = 0.0001$; HC versus GUA: $P < 0.0001$), with oHC exhibiting a significantly better survival rate than HC ($P = 0.0014$) (Fig. 1C). We then compared the longevity of female adults when they were provided with only water but not sugar. The results showed that both oHC and HC females had significantly shorter lifespans than GUA females (median survival days, oHC: 5 days; HC: 4 days; GUA: 6 days; all $P < 0.0001$), and oHC exhibited significantly longer lifespan than HC ($P < 0.0001$) (Fig. 1D). As *A. albopictus* overwinters through egg diapause induced by a short-day photoperiod in most areas of Chinese mainland, including Guangzhou, we compared the ability to induce egg diapause of the three mosquito strains under artificial conditions for diapause induction. The results showed no difference in egg hatch rate under a long-day photoperiod [Fig. 1E, $F (2, 6) = 1.018, P = 0.42$]. However, under a short-day photoperiod, both oHC and HC eggs had significantly higher hatch rates than GUA eggs (oHC: 43.37 ± 1.03%; HC: 53.95 ± 0.39%; GUA: 11.99 ± 0.36%; all $P<0.0001$), with oHC having a lower egg hatch rate than HC ($P = 0.0002$) (Fig. 1E). We further distinguished between diapause and dead eggs in those unhatched eggs. Both oHC and HC eggs exhibited significantly lower diapause rates than GUA eggs (oHC: 68.00 ± 0.83%; HC: 53.33 ± 4.91%; GUA: 96.67 ± 1.36%; oHC versus GUA: $P = 0.0035$; HC versus GUA: $P = 0.0004$), while no significant difference in diapause rates was observed between oHC and HC ($P = 0.0662$) (Fig. 1F). Finally, we calculated the incidence of overwintering, defined as the proportion of diapause eggs laid by females post diapause induction. The results showed that both oHC and HC had significantly lower incidence of overwintering compared to GUA (oHC: 38.50 ± 0.65%; HC: 24.59 ± 2.41%; GUA, 85.07 ± 1.07%; all $P < 0.0001$), with oHC having a significantly higher incidence of overwintering than HC ($P = 0.0052$) (Fig. 1G).

### Model of population suppression by a standalone IIT with population replacement prevented

**Fig. 2:**
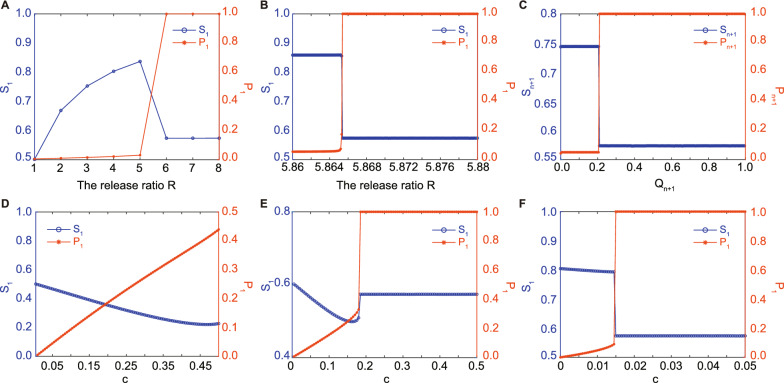
Modelling the population suppression by a standalone IIT using the *A. albopictus* HC mosquito with population replacement prevented. **A** The nonlinear dependence of the annual suppression efficiency $S_1$ and the infection frequency $P_1$ on the constant release ratio $R\in [1, 8]$ at the fixed female HC contamination rate $c=0.5\%$. The standalone IIT strategy can suppress up to 83% of wild mosquitoes with an annual infection frequency $P_1=3.07\%$ when $R=5$. **B** The enlarged curves in the tiny interval $R\in [5.86, 5.88]$. $S_1$ decreases from 85.86 to 57.37%, while $P_1$ increases from 5.44 to 100%. A heavier release of HC males with $R>5$ brings a high risk of population replacement. **C** The dependence of the suppression efficiency $S_{n+1}$ and the infection frequency $P_{n+1}$ at the end of the hot season on the infection frequency at the beginning of the hot season $Q_{n+1}$, which should stay below 20.5% to prevent population replacement. The maximal tolerance of HC female contamination without causing population replacement, with the release ratios *R* of HC to GUA being 1 (**D**), 1.5 (**E**), and 3.92 (**F**) to target for 50, 60, and 80% population suppression in the field, respectively. **D** When the release ratio $R\le 1$, the mosquito population will not be completely replaced even if released mosquitoes are not sexually sorted, and the maximal infection rate is about 43.95%. The curve $S_1$ decreases in *c* linearly, while $P_1$ increases in *c* almost Linearly. If we increase the release ratio to 1.5 to pursue 60% suppression of wild mosquitoes, the maximal tolerance of HC female contamination is about 18% (**E**). Different from (**D**), the larger release ratio in **E** makes $S_1$ not monotonic for $c\in [0.5\%, 18\%]$: it undergoes a slight reduction from 60 to 49.86% when *c* goes from 0.5 to 16%, and then increases to 50.91% when *c* hits the maximal tolerance rate 18%. **F** Pursuing 80% suppression of wild mosquitoes drags the maximal tolerance of HC female contamination down to 1.45%

We thus conducted mathematical simulations (details are described in the Supplemental information) to predict the population dynamics following a standalone IIT operation in the field, taking into account the above effects of adverse field conditions on HC mosquitoes, together with complete CI and a perfect maternal transmission rate (Fig. S1). We also considered a 0.5% HC female contamination rate, achievable with the currently widely used mechanical sex separation. The results from our mathematical model suggest that the standalone IIT strategy could potentially suppress up to 83% of wild mosquitoes, with an annual infection frequency of 3.07% when the release ratio is set at 5, which is the maximum level without triggering population replacement (Fig. 2A). A slight further increase in the release ratio could potentially lead to population replacement and reduce suppression efficiency. Indeed, when the release ratio falls within the narrow range of [5.86, 5.88], the projected suppression efficiency decreases from 85.86% to 57.37%, and the annual infection frequency rapidly rises from 5.44% to 100% (Fig. 2B). Furthermore, the model suggests that even with HC population replacement, about 60% suppression is maintained instead of population restoration, mainly due to the substantial fitness cost associated with adverse environmental conditions. When we factor in the reduced overwintering ability of HC eggs during the cold seasons compared to GUA eggs, we observe that by releasing HC mosquitoes at a constant ratio of 3 and a contamination rate of $\le$ 1.5%, as long as the infection frequency at the start of mosquito season remains below 20.9%, the mosquito population can be effectively suppressed. This leads to an annual suppression efficiency of 74.42% and a moderate infection frequency of 5.05% (Fig. 2C). In IIT, the population’s ability to tolerate female contamination depends on release ratios or suppression efficiency. When the release ratio of HC to GUA is 1 or less, or if the goal is to suppress only 50% or less of wild mosquitoes, the mosquito population won’t be entirely replaced, even if no sexual sorting of released mosquitoes is done (Fig. 2D). When aiming for an 60% suppression of wild mosquitoes with the ratio 1.5 of HC to GUA, the maximum tolerance for HC female contamination without causing population replacement is approximately 18% (Fig. 2E). For an 80% suppression target of wild mosquitoes with the ratio 3.92 of HC to GUA, the maximum tolerance for HC female contamination decreases to 1.45% (Fig. 2F).

### Effective population suppression maintained by a standalone IIT with low release doses

**Fig. 3:**
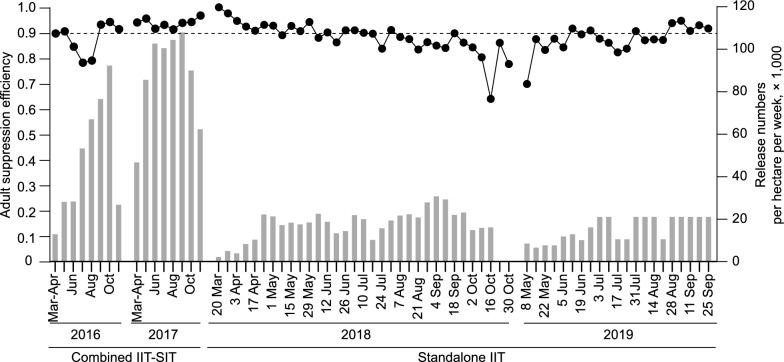
The *A. albopictus *HC male release numbers and adult suppression efficiency from 2016 to 2019 on Shazai Island. The grey bars represent weekly male release numbers, and the black dots represent the suppression efficiency of female *A. albopictus*. The data for 2016 and 2017 are displayed monthly, while the data for 2018 and 2019 are presented weekly. A standalone IIT was implemented in the field to suppress the mosquito population from 2018 to 2019, whereas a combined IIT–SIT approach was applied in 2016 and 2017. $n = 36$ weeks in 2016 and 2017, $n = 33$ weeks in 2018, $n = 21$ weeks in 2019

**Fig. 4:**
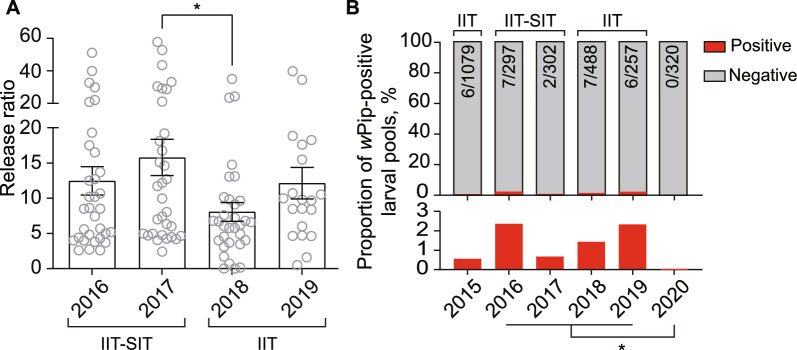
A standalone IIT with low-dose release maintains consistent high release ratios and does not result in population replacement. **A** The average yearly release ratio from 2016 to 2019. Mean ± SEM, and significance were determined by two-tailed Mann Whitney U test, $*P= 0.016, n = 33$ weeks in 2016, $n = 32$ weeks in 2017 and 2018, $n = 20$ weeks in 2019. **B** The temporal distribution of *w*Pip-positive larvae pools on Shazai Island for six consecutive years. The proportion of *w*Pip-positive and negative larvae pools is represented by red and gray bars, respectively. The lower part of the figure shows a magnified view of the proportion of *w*Pip-positive larval pools. The number of *w*Pip positive pools and total larvae pools tested are listed above each bar. The *w*Pip positive rate had no significant difference from 2016 to 2019. Fisher’s exact test, all $P> 0.05$. Following the cessation of mosquito releases at the end of 2019, there was a significant decrease in the number of *w*Pip-positive larvae pools collected from Shazai Island throughout 2020. Fisher’s exact test, $* P = 0.013$

Unlike the previous report [21], we weekly released a low number of non-irradiated HC males to explore both the minimum release dose required to maintain suppression and the potential to prevent population replacement. The female contamination rates remained consistent with those reported in 2016–2017 [21], and the average female contamination rate was approximately 0.27%. The *A. albopictus* population size was monitored by using BG traps (see methods and Fig. S2). The results showed a yearly adult suppression efficiency of 88.3% and 87.7% with an average release number of approximately 16,500 and 15,400 per hectare per week in 2018 and 2019, respectively (Fig. 3 and Fig. S3). These suppression efficiencies were similar to those achieved in the previous combined IIT-SIT field trial, where yearly adult suppression efficiencies were 87.8% and 93.9% with release numbers of 48,350 and 86,900 per hectare per week in 2016 and 2017, respectively. Consistently, even with the reduced release numbers, the average release ratios in 2018 and 2019 were 8.07 and 12.13, respectively (Fig. 4A), well above the required critical overflooding ratio of 5 [21]. Compared to the observed release ratios, 12.47 and 15.79, in 2016 and 2017, respectively, the only significant difference was between 2017 and 2018 (Fig. 4A, $P = 0.02$). These results demonstrate that IIT effectively maintains population suppression, even with an 82.3% reduction in the number of released males.

To assess the risk of population replacement, we monitored the *w*Pip infection frequency in larvae collected via ovitraps. In 2018 and 2019, a total of 7 and 6 ovitraps (larvae pools) were found to contain *w*Pip-positive larvae out of 488 and 257 larvae pools, respectively (Fig. 4B). Notably, the proportion of *w*Pip-positive ovitraps, which was 1.43% in 2018 and 2.33% in 2019, showed no significant difference from the rates observed in previous years (2.36% in 2016 and 0.66% in 2017) ($P> 0.05$). As previously observed, *w*Pip-positive locations were isolated both spatially and temporally (Fig. S4). Moreover, in 2020, when mosquito releases were discontinued, none of the 320 larvae pools collected from Shazai Island throughout the year were *w*Pip positive (Fig. 4B, $P = 0.013$). In addition, during three human landing catches conducted across mosquito seasons, a total of 205 females were collected at 58 locations (Fig. S5), and none of them tested positive for *w*Pip.

### Impact of IIT-driven suppression on target and non-target mosquito species

**Fig. 5:**
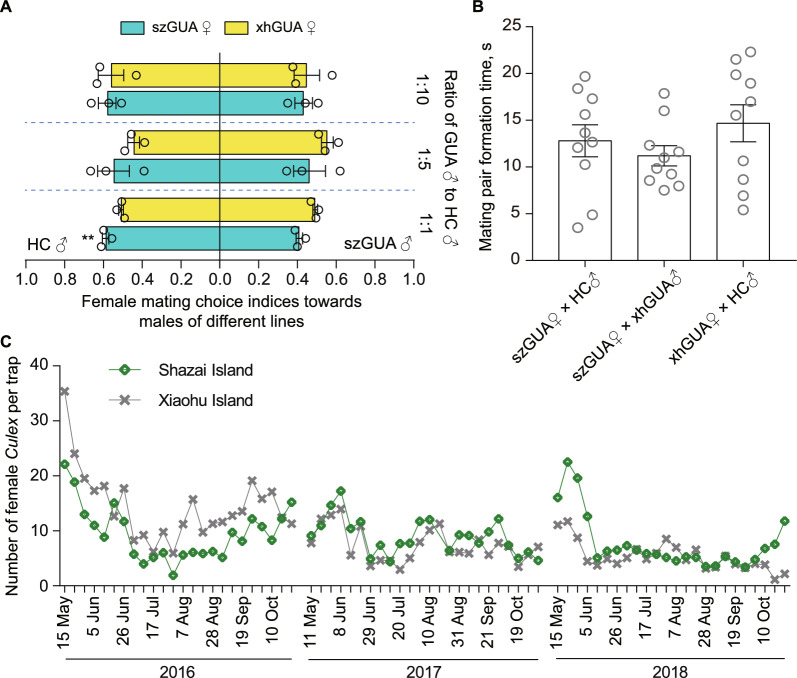
The impact of 3 year suppression on target and non-target mosquito species. **A** *A. albopictus* female mating choice indices were compared between Shazai and Xiaohu females under different male release ratios. Three replicates were conducted for each release ratio. Individuals collected from Shazai island or the non-release control site, Xiaohu island, are denoted as “sz” or “xh”, respectively, in front of wild “GUA” mosquito. Mean ± SEM and significance were determined by a two-tailed *t* test, $$** P < 0.01$$. Experimental design and data are listed in Tables S1 and S2. **B** Comparison of times in forming mating pairs when field-derived *A. albopictus* females encounter mating attempts from HC males or field-derived males. Across all three mating groups, there were no significant differences in mating pair formation times. Mean ± SEM and significance were determined by one-way ANOVA. $$n = 10$$ for each group, $$P = 0.34$$. **C** The abundance of *C. quinquefasciatus*, as a non-target mosquito species, in release (Shazai) and non-release (Xiaohu) areas across three years. The average number of female *C. quinquefasciatus* per BG trap was recorded weekly from 2016 to 2018. The overall data collected from this period indicates that there was no significant difference in the population size of *Culex* between Shazai and Xiaohu islands during the *A. albopictus* suppression period. Two-tailed Mann Whitney U test, $$n = 72$$ weeks, $$P = 0.796$$. In 2016, the *Culex* population size on Shazai Island was significantly smaller compared to the control site, while no significant difference was observed in 2017 and 2018. Two-tailed Mann Whitney U test, 2016, $$n = 24$$ weeks, $$P = 0.002$$; 2017, $$n = 24$$ weeks, $$P = 0.095$$; 2018, $$n = 24, P = 0.106$$. The correlation in the population size of *C. quinquefasciatus* between Shazai and Xiaohu Islands was compared by the Pearson correlation test, $$r = 0:61$$, $$n = 71$$, and $$P < 0:0001$$

Strong population suppression over an extended period in the field may select for target females to develop resistance to mating with HC males, while long-term mass-rearing may alter the mating behavior of HC males due to laboratory adaptation. This prompted us to investigate whether the mating preferences of residual wild females on Shazai Island had changed after experiencing IIT suppression for over three years, causing them to be reluctant to mate with our released HC males. A dual-choice mating experiment was then conducted (Table S1). Based on the results of 747 blood-taken females collected from the cages, we did not reveal any decrease in the mating choice of Shazai females toward HC males as compared to Xiaohu females at all three ratios (Fig. 5A and Table S2). Interestingly, a higher female mating choice to HC males was observed in Shazai females at a GUA vs HC ratio of 1∶1 ($P = 0.0016$), suggesting that more Shazai females chose to mate with HC males than Shazai males. In a second experiment, we directly assessed the strength of female mating preferences by measuring how quickly females make a choice when Shazai females were provided with the option to mate with either HC or Xiaohu males. The results showed no significant difference in the time it took for mating pairs to form when Shazai females mated with HC males compared to control groups in which Shazai females mated with Xiaohu males or Xiaohu females mated with HC males (Fig. 5B, $P = 0.34$). Thus, Shazai females had not displayed any reduced capacity to mate with HC males in terms of both choice frequency and time to choice.

We investigated whether strong suppression of *A. albopictus* over an extended period in Shazai Island would lead to an increase in the population of non-target *C. quinquefasciatus*, which was reported to share breeding habitats with *A. albopictus* [43]. The results showed that there was no significant difference in the population size of *C. quinquefasciatus* between Shazai and Xiaohu Islands during the years 2016 to 2018 when *A. albopictus* was strongly suppressed in Shazai Island (Fig. 5C, $P = 0.796$). We also observed a strong correlation in the population size of *C. quinquefasciatus* between Shazai and Xiaohu Islands across these three years ($P < 0.0001$), Likely due to the very similar ecological environments shared by these two locations. Data for the year 2019 were not included due to significant environmental changes resulting from residential community renovations in Xiaohu Island. When comparing the data by year, there were no significant differences between the two locations, except for a lower population observed in Shazai in 2016 (2016, $n = 24, P = 0.002$; 2017, $n = 24, P = 0.095$; 2018, $n = 24, P = 0.106$). This reduction was likely due to the efforts to gain community support for the field trial by removing breeding sites in Shazai.

### Population rebounding gradually after release stopped

**Fig. 6:**
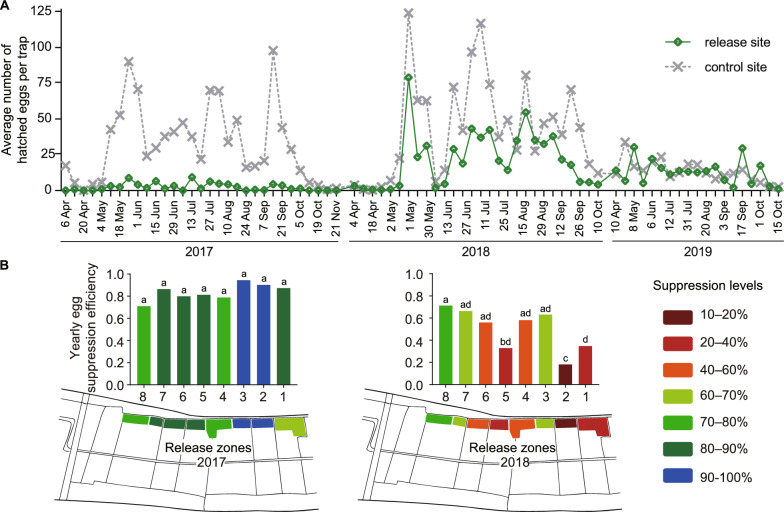
The recovery dynamics of field *A. albopictus* population after HC male mosquito releases stopped. **A** The average number of hatched eggs per trap from 2017 to 2019. Mosquito release activity was stopped at the end of 2017 in Dadaosha island, but the field *A. albopictus* population was continually monitored using ovitraps in 2018 and 2019. As compared to the control site, the release site had a significantly smaller *A. albopictus* population size in 2018, while the difference disappeared in 2019. Two-tailed Mann Whitney U test, 2018, $n = 28, P = 0.013$; 2019, $n = 19, P = 0.693$. **B** The spatial dynamics of yearly egg suppression efficiency across different zones in 2017 and 2018. The data revealed significant variations in the recovery levels of the mosquito populations. Specifically, zones adjacent to the non-release area (zone 1 and 2) and zones with higher levels of human trafficking (zone 5) exhibited a significantly higher level of mosquito population recovery. Monthly average eggs per trap of each zone were compared and analyzed by Paired *t*-test, $n = 30$ in 2017, $n = 28$ in 2018. Different letters above each column indicate significant differences, $P < 0.05$

To evaluate how the mosquito population rebounded after the period of population suppression, we halted the release of HC males on Dadaosha Island at the end of 2017 following two consecutive years of strong *A. albopictus* suppression [21], and then weekly monitored the *A. albopictus* population using ovitraps (Fig. S6). In 2018, we observed a gradual increase in the *A. albopictus* population on Dadaosha Island, as evidenced by the number of hatched eggs per trap. This population rebounded to approximately half the size of the control site (Fig. 6A, $P = 0.013$). In 2019, there was no significant difference in population size between the two sites ($P = 0.693$), indicating that the population might recover fully within a year after the cessation of releases. However, it is worth noting that both sites showed significantly lower mosquito population sizes in 2019 relative to 2018 ($df = 2, F = 5.852, P = 0.004$), as a result of local government initiatives targeting the enhancement of the environment under the “beautiful countryside” program, which involved rigorous mosquito control measures such as environmental management and insecticide application.

We conducted a further analysis of the spatial dynamics of population recovery across eight zones within Dadaosha Island (Fig. 6B) from 2017 to 2018. Notably, mosquito populations in zones 1 and 2, which are closer to the non-release area, exhibited a faster recovery, as evidenced by having lower yearly egg suppression efficiency (34.71% and 18.12% in zone 1 and 2, respectively). In contrast, zones 3, 4, 6, 7, and 8, which are further from the non-release area, maintained better population suppression, indicating the influence of mosquito migration on the rapid population recovery (suppression efficiencies, zone 3: 63.13%, zone 4: 58.12%; zone 6: 56.05%; zone 7: 66.30%; zone 8: 71.32%). Zone 5 also experienced a swift population rebound (suppression efficiency: 32.98%), possibly due to its proximity to a shopping store (the sole store in this area) that fosters increased human activities and mosquito migration.

## Discussion

Previous studies have indicated that *Wolbachia* transinfection and/or long-term laboratory adaptation can significantly reduce mosquito survivability under adverse conditions [44, 45]. Given the decreased survival ability of *Wolbachia* transinfected mosquitoes can potentially mitigate the risk of population replacement during an IIT project, we therefore examined HC mosquito fitness under adverse field conditions. As HC differs from the field GUA in both *Wolbachia* infection and potential genetic background changes from adaptation to our mass-rearing facility, we first outcrossed the HC line with the newly field-collected GUA line for four generations to generate the oHC line with homogeneous genetic backgrounds. Comparisons between oHC and HC and between oHC and GUA would enable us to determine the contribution of genetic background and *Wolbachia*, respectively, to a tested trait. We observed a significant reduction in stress resistance in both oHC and HC mosquitoes, specifically in terms of larval survival under malnutrition, high temperatures, and female longevity under stress. These results indicate both *Wolbachia* transinfection and long-term laboratory adaptation can indeed reduce the stress resistance of HC mosquitoes. Our field sites, situated in subtropical regions with distinct seasonality, experience low temperatures and low precipitation during the winter, which is unfavorable for *A. albopictus* development. Consequently, these mosquitoes produce diapause eggs for overwintering [39]. In our study, we observed that both oHC and HC females exhibited significantly impaired diapause egg production ability. This raises the possibility that even if HC mosquitoes establish a small population during warm and rainy seasons, their survival through the winter is doubtful. Furthermore, our HC mosquitoes were probably susceptible to insecticides, as any potential insecticide resistance is likely lost during long-term rearing [46]. Consequently, the application of insecticides in release areas could also potentially eliminate HC mosquitoes. Recent research has suggested that achieving population replacement with insecticide-sensitive females in the field is challenging [47]. These results encouraged us to perform a standalone IIT field trial based on releasing HC males in Guangzhou City.

Our study, both mathematically and experimentally, demonstrates the effectiveness of low-dose standalone IIT based on releasing HC males in maintaining high population suppression without invoking population replacement. During our maintenance release phase, we only required about 20% of the initial release number, primarily to manage a small quantity of local residual and immigrated mosquitoes from untreated areas. In addition to reducing the release number, we optimized the release frequency in 2018 and 2019 to twice a week compared to the previous three times a week. The release ratio was determined by directly assessing mosquito captures from BG traps rather than the costly and time-consuming PCR detection (see methods). These findings suggest that in the context of a long-term application of IIT-based mosquito management program, following the initial suppression of mosquito populations, there is potential to significantly decrease the number of released mosquitoes, transportation time, release frequency, and monitoring intensity during the maintenance phase of population suppression. This optimization of operational parameters is expected to result in substantial cost savings. Additionally, our results suggest that under adverse field conditions, such as extended period of drought, heat weaves, or low temperatures, *Wolbachia*-associated fitness cost may significantly reduce the risk of population replacement during IIT application. Consequently, it is unnecessary to use the costly irradiators or AI-based mosquito sex-separation systems, further enhancing the cost-effectiveness and accessibility of IIT. However, it is important to acknowledge that in scenarios targeting population elimination or in regions with year-round favorable climates for mosquito growth, the combination of irradiation or the development of precise mosquito sex-separation systems remains essential. Given that the mosquito fitness costs associated with *Wolbachia* infection are often strain-specific, the adoption of a standalone IIT should be assessed individually, considering the unique circumstances of each case. Our studies emphasize the need for evaluation of the fitness of the transinfected line under realistic field conditions and, by extension, the fitness of all mosquitoes intended for population suppression or replacement should be assessed in their target field environments.

We leveraged the *A. albopictus* suppression on Shazai Island to study potential changes in female mating preference. This area had seen wild-type *A. albopictus* females under intense mating pressure from a large number of HC males over multiple years. We compared the frequency of females choosing HC males versus Shazai males, as well as the decision-making time between Shazai and Xiaohu females, who have not been exposed to the males’ release. Consequently, we did not observe evidence supporting the development of female mating preference following 3-year population suppression. On the one hand, it may take much longer time to evolve this mating behave change. On the other hand, it is also worthy to note that our experiment was conducted in laboratory cages and mass-reared HC males are likely to mate well under lab conditions in an enclosed space so their apparent success may be inflated. If it occurs, this advantage may mask our ability to detect the female mating preference as the outcome of mating is determined by both female mating preference and male mating competitiveness. In the field, mating behavior could be influenced by ecological factors such as variable male densities, spatial distribution, environmental cues (e.g., vegetation, temperature, humidity), and competitive interactions. Because these variables were absent from our cage-based experiments, mating dynamics in the wild may differ. To improve generalizability, future studies should incorporate field observations or large-scale enclosures to validate our findings under realistic conditions, highlighting the need for complementary field experiments to confirm the robustness of mating preference results in natural settings.

As expected, mosquito population gradually rebounded after release stopped. The rate of mosquito population recovery varies depending on factors such as the degree of isolation of the release area and the extent of population suppression. Our study, in line with other field studies employing IIT strategies [20] showed that the wild mosquito population did not rebound rapidly after the release ceased. Instead, the suppression effect persisted for an extended duration. In our study site, Dadaosha Island, the suppression of the *A. albopictus* population lasted for a year, with the annual population size only rebounding to approximately 50% of its pre-suppression levels in the control site. The prolonged suppression effect indirectly indicates that the potent reduction of the wild population results in depleting the egg bank at the release site. Although its population appeared to recover fully in Year 2 after the cessation of releases, we cannot rule out the possibility that its population size remained lower compared to the control site. This uncertainty arises due to the rigorous mosquito control ongoing in these areas in 2019. Moreover, our spatial population dynamic analyses, consistent with previous studies [19, 21], suggest that the degree of site isolation is an important determinant of recovery rates, largely through its effect on mosquito immigration. Our case study further demonstrates that even in relatively isolated locations, population recovery can be accelerated by human mobility, highlighting the role of human movement in facilitating mosquito immigration. Nevertheless, without dedicated spatial and demographic approaches, the relative contributions of immigration and local control measures to rebound dynamics remain unresolved.

Finally, it is important to recognize that our results may not be fully generalizable, as they were shaped by the island setting, the subtropical climate of Guangzhou, and the particular transinfected *A. albopictus* strain examined. More complex urban environments, with higher mosquito densities and more dynamic immigration patterns, may require adapted release strategies, which could limit the direct transferability of our findings. In addition, although our suppression efforts necessarily reduced opportunities for mosquito–human contact, direct epidemiological outcomes such as changes in dengue incidence could not be assessed because no local cases occurred during the trial period. Future studies should extend these findings by evaluating standalone IIT across diverse ecological contexts, integrating disease surveillance endpoints, and exploring adaptive approaches to optimize large-scale urban implementation.

## Conclusions

Our study demonstrates that field populations can tolerate a certain level of female contamination in IIT, attributable to *Wolbachia*-associated fitness costs under adverse field conditions and other disadvantages linked to long-term mass rearing. This finding enables IIT to effectively suppress mosquito populations using the current mechanical sex sorter without triggering population replacement. The population suppression can be maintained with only 20% of the initial release dose. Population gradually rebounds after release stops with the speed of recovery likely influenced by mosquito immigration associated with population flow. We have neither detected the development of female mating preference to avoid mating with HC males nor observed an impact on non-target *Culex* species after three years of suppression, although further studies are needed to validate these findings. Our future research will focus on integrating population suppression and population replacement to develop an optimal mosquito control strategy to prevent disease outbreaks and identifying the most suitable *Wolbachia* strain for this integration under varying environmental conditions.

## Supplementary Information

Supplementary material 1.

## Data Availability

All data are available in the main text or the supplementary materials.

## Abbreviations

IIT: Incompatible insect technique
SIT: Sterile insect technique
CI: Cytoplasmic incompatibility
HC: *Aedes albopictus* HC line
GUA: Wild-type *Aedes albopictus* line
oHC: Outcrossed HC line
FMC: Female mating choice
BG traps: Biogents traps
ANOVA: Analysis of variance
SEM: Standard error of the mean
