# Supplementary material for: Ecological dynamics of field Aedes albopictus populations under Wolbachia-mediated suppression
Source: Infect Dis Poverty. 2025 Sep 24;14:96. doi: 10.1186/s40249-025-01367-9 (PMC12459051; doi:10.1186/s40249-025-01367-9)
Supplement: Supplementary file 1 — Supplementary material 1. [file 40249_2025_1367_MOESM1_ESM.pdf]

Supplemental Materials and Methods, Tables S1-S2, and Figures S1-S6

Mathematical Modeling

Mathematical Background

The abundance of *A. albopictus* in Guangzhou shows almost the same yearly growth pattern [1–3]. Female *A. albopictus* mosquitoes in Guangzhou lay diapause eggs in the middle of October, similar to those in the temperate zones of Europe and North America [4]. The hatching of these diapause eggs in early March leads to the first peak of adult population size in late May or early June. Due to the density-induced larval death in hot summer, the population size decreases for one to two months and then bounces back and reaches the second peak in September or October due to the moderate temperature in the autumn. Then, the abundance sharply declines and eventually vanishes in dry winter since eggs enter a diapause state again. To assess the impact of the significantly reduced overwintering ability of HC eggs relative to GUA eggs on population suppression, we divide each year into two seasons: the hot season from March 1 to October 15, and the cold season from October 16 to the end of next February. The mosquito release season coincides with the hot season, and there is no mosquito release in the cold season. We let $n = 1, 2, \cdots$ denote years, and t_n^0 denote March 1 in Guangzhou, the start date of mosquito release. Let t_n^1 be October 15, the end date of mosquito release. We simulate

the population dynamics of adult mosquitoes in the hot season $[t_n^0, t_n^1]$ by developing a model of ordinary differential equations. For the cold season $[t_n^1+1, t_{n+1}^0-1]$, we simulate the decay of egg numbers through an elementary mathematical approach since there is no active reproduction of adult mosquitoes.

Population Dynamics of Adult Mosquitoes in the Hot Season

We begin by discussing the population growth of adult GUA mosquitoes in the wild area without the interference of HC mosquito releases. Let $x(t)$ be the number of adult GUA females/males on day t. Let b_1 be the number of adult female offspring per GUA female per day, and δ_1 the density-independent death rate of adult GUA females. Naturally,

$$b_1 > \delta_1. \tag{1}$$

The population dynamics of adult GUA females during the hot season is modeled by the canonical Logistic equation

$$x'(t) = b_1 x(t) - \delta_1 \left(1 - \frac{x(t)}{K}\right) x(t) = \frac{\delta_1}{K} x(t) \cdot \left[\frac{(b_1 - \delta_1)K}{\delta_1} - x(t)\right]. \tag{2}$$

When (1) holds, we see that equation (2) admits a trivial equilibrium point $x(t) \equiv 0$, and a unique positive equilibrium $x^* = \mathcal{K} := \dfrac{(b_1 - \delta_1)K}{\delta_1}$. Equation (2) yields $x'(t) > 0$ when $x(t) \in (0, \mathcal{K})$ and $x'(t) < 0$ when $x(t) > \mathcal{K}$. It shows that $x = 0$ is unstable, and x^* is globally asymptotically stable [5]. We call $\mathcal{K}$ the carrying capacity of adult GUA mosquitoes since any positive solution $x(t)$ of (2) approaches $\mathcal{K}$ as $t \to \infty$.

Next, we extend equation (2) to incorporate the release of HC mosquitoes in wild areas. Let R be the release ratio of HC mosquitoes, including HC males and accidentally released HC females, to wild-type males. Hence, the total number of released HC mosquitoes equals $Rx(t)$. Let c be the HC female contamination rate. Then the number of HC females released is $cRx(t)$, and the number of HC males released during the hot season is $(1-c)Rx(t)$. Let $y(t)$ be the number of adult HC females/males. Under random mating behavior, the production rate decreases from b_1 to

$$b_1 \cdot \frac{x(t)}{[(1-c)R+1]x(t)+y(t)}$$

due to complete CI. Also, the density dependence rate increases from $\delta_1 x(t)/K$ to

$$\delta_1 \frac{x(t)+y(t)}{K},$$

due to competition between adult females. Hence, the dynamics of GUA females obey

$$x'(t) = \frac{b_1 x^2(t)}{[(1-c)R+1]x(t)+y(t)} - \delta_1 x(t)\left(1+\frac{x(t)+y(t)}{K}\right). \tag{3}$$

Regarding the number of HC females, we should include the accidental release of HC females in their growth. Since HC females produce viable offspring, regardless of the infection status of their mating partner, we introduce

$$y'(t) = b_2 \cdot [y(t)+cRx(t)] \cdot \left(\frac{y(t)}{x(t)+y(t)}\right)^{\alpha} - \delta_2 y(t)\left(1+\frac{x(t)+y(t)}{K}\right), \tag{4}$$

to characterize the dynamics of HC females, where b_2 is the number of offspring per female per day, δ_2 is the density-independent death rate, and α estimates the frequency-dependent competition pressure of HC mosquitoes in the wild area due to the competition

with GUA mosquitoes. Equations (3) and (4) constitute the model for the interactive dynamics of GUA and HC mosquitoes during the hot season.

At the end of the hot season, the suppression efficiency is quantified by the relative decrease in female mosquitoes

$$S_n = \frac{\mathcal{K} - x(t_n^1) - cRx(t_n^1) - y(t_n^1)}{\mathcal{K}}, \tag{5}$$

and the infection frequency is given by the proportion of HC females among all females

$$P_n = \frac{y(t_n^1) + cRx(t_n^1)}{x(t_n^1) + y(t_n^1) + cRx(t_n^1)}, \tag{6}$$

where $n = 1, 2, \cdots$.

Population Dynamics of Eggs in the Cold Season

Let $\bar{x}(t)$ and $\bar{y}(t)$ be the number of GUA and HC eggs, respectively. The overwintering ability of GUA and HC eggs is characterized by the probability that an egg survives the cold season. Let $d_G, d_H \in (0, 1)$ be the incidence of overwintering of GUA eggs and HC eggs, respectively. Then we have

$$\bar{x}(t_{n+1}^0) = d_G \bar{x}(t_n^1),\ \bar{y}(t_{n+1}^0) = d_H \bar{y}(t_n^1),\ n = 1, 2, \cdots, \tag{7}$$

The suppression of GUA mosquito population size at the end of the hot season is reflected by a decrease in mosquito numbers at the beginning of next year. The degree of this decrease is quantified by the suppression efficiency in the following simple equality:

$$x(t_{n+1}^0) = (1 - S_n) \cdot x(t_n^0),\ n = 1, 2, \cdots, \tag{8}$$

where S_n is defined in (5). Regarding $y(t^0_{n+1})$, we bridge the number of eggs and adults with the relation that the ratio of egg numbers to adult numbers is the same for GUA and HC. That is, $\bar{x}(t^0_{n+1}) : x(t^0_{n+1}) = \bar{y}(t^0_{n+1}) : y(t^0_{n+1})$. Therefore, by using (6)-(8), we have

$$\frac{y(t^0_{n+1})}{x(t^0_{n+1})} = \frac{\bar{y}(t^0_{n+1})}{\bar{x}(t^0_{n+1})} = \frac{d_H \bar{y}(t^1_n)}{d_G \bar{x}(t^1_n)} = \frac{d_H[P_n - cR(1-P_n)]}{d_G(1-P_n)},$$

and the HC females in the next hot season initiate at

$$y(t^0_{n+1}) = \frac{d_H[P_n - cR(1-P_n)]x(t^0_{n+1})}{d_G(1-P_n)} = \frac{d_H[P_n - cR(1-P_n)](1-S_n)x(t^0_n)}{d_G(1-P_n)}, \quad (9)$$

where $n = 1, 2, \cdots$.

The Parameter Values and the Initial Data for Simulation

Estimating δ_1 and δ_2. Assume that the number $x(t)$ of surviving GUA females obeys the differential equation $x'(t) = -\delta_1 x(t)$, whose general solution is $x(t) = x(0)\exp(-\delta_1 t)$. Let h_G be the half-life at which the initial population size is reduced to half. Then $x(0)/2 = x(0)\exp(-\delta_1 h_G)$, from which we obtain the conversion formula $\delta_1 = \log(2)/h_G$. Similarly, we get $\delta_2 = \log(2)/h_H$, where h_H is the half-life of HC females. Since $h_G = 6$ and $h_H = 5$, we get

$$\delta_1 = 0.1155, \ \delta_2 = 0.1386.$$

Estimating b_1 and b_2. It is a formidable task to count the number of offspring per mosquito per day, b_1, which has the largest variation. From Table 6 in [3], the fecundity per mosquito is seasonally dependent, ranging from 28.36 to 224.5. And the probability

for an egg to survive to larva, pupa, and finally emerge to an adult ranges from 4/1000 to 337/1000, depending on the nutritional status of habitats. This leads to

$$b_1 \in [28.36 \times 4/1000, 224.5 \times 337/1000] = [0.1134, 75.6565].$$

Fitness cost of oHC mosquitoes compared to GUA mosquitoes. oHC larvae had significantly lower survival rates under malnourished conditions (GUA: 82.3± 1.19%, oHC: 67.0±1.7%) and high cyclical temperatures (GUA: 74.8±2.9%, oHC: 47.60±2.55%). These data suggest $f_1 = 67/82.3$, $f_2 = 47.6/74.8$. To estimate the fitness cost of oHC compared to GUA, we set $b_2 = fb_1$ with $f = f_1 \times f_2 \approx 0.5181$ for oHC mosquitoes. The parameters and the initial conditions are listed in the following Table.

Numerical Findings

Up to 83% of wild mosquitoes can be suppressed at our current HC female contamination rate 0.5% with no risk of population replacement. By inserting the parameter values and the initial conditions specified in Table 1 into equations (3) and (4), we calculate the suppression efficiency S_1 and the infection frequency P_1 at the end of the first hot season with $R \in [1, 8]$. Figure 2A manifests that S_1 increases in R along a smooth and concave curve, and P_1 increases in R along a convex curve when $R \leq 8$, with $S_1 \in [0.5010, 0.8369]$ and $P_1 \in [0.0050, 0.0307]$. Such a growth trend is suddenly disrupted when R is increased beyond 5. The curve of P_1 displays a striking feature in that it jumps from 3.07% to nearly 100% rather quickly, and the suppression efficiency S_1 falls to about 57.37% when the infection frequency reaches 100%. Figure 2B indicates

Table 1: Parameter values and initial conditions for equations (3) and (4).

Description	Notation	Value
Number of offspring per GUA female per day	b_1	12 (Fitted)
Number of offspring per oHC female per day	b_2	6.2172
Overwintering ability of oHC eggs	d_H	0.3850
Overwintering ability of oHC eggs	d_G	0.8507
Density-independent death rate of GUA females	δ_1	0.1155
Density-independent death rate of oHC females	δ_2	0.1386
Contamination rate in 2018 and 2019	c	0.5%
Infection frequency competition intensity	α	0.5 (Fitted)
The unique positive equilibrium of (2)	$\mathcal{K}$	1000
Carrying capacity parameter	K	$K = \dfrac{1000\delta_1}{b_1 - \delta_1} \approx 9.7185$
Initial value of GUA females at the 1-st year	$x(t_1^0)$	$\mathcal{K}$
Initial value of oHC females at the 1-st year	$y(t_1^0)$	$cRx(t_1^0)$
Initial value of GUA females at the $(n+1)$-st year	$x(t_{n+1}^0)(n \geq 1)$	(8)
Initial value of oHC females at the $(n+1)$-st year	$y(t_{n+1}^0)(n \geq 1)$	(9)

that within the short interval $R \in [5.86, 5.88]$, S_1 decreases from 85.86% to 57.37%, while P_1 increases from 5.44% to 100%. The suppression efficiency 57.37%, expected to be 0 with complete population replacement at $R = 5.88$, is due to the fitness cost of infected females, which results in a smaller mosquito population size. To check it numerically, we calculate

$$x(t_n^1) = 0, \; y(t_n^1) = 426.2646 < \mathcal{K} = 1000,$$

when $R = 5.88$, and hence $S_1 = 1 - \frac{426.2646}{1000} = 57.37\%$ and $P_1 = 1$.

By examining the numerical data further, we claim that the standalone IIT strategy can suppress up to 83% of wild mosquitoes with an annual infection frequency $P_1 = 3.07\%$ when $R = 5$. A heavier release of HC males with $R > 5$ is not recommended because it brings a high risk of population replacement. Indeed, we find it more optimal to release fewer HC mosquitoes. For instance, we suggest a release ratio $R = 3$, which is sufficient to suppress about 75.27% wild mosquito population with a small infection frequency $P_1 = 1.55\%$.

Dependence of the suppression and infection of mosquitoes at the end of the hot season on the infection frequency at the beginning of the hot season. We use equations (3) and (4) to examine how the infection frequency at the beginning of the hot season affects the suppression and infection of mosquitoes at the end of the hot season. At the beginning of the hot season of year $n+1$, let $Q_{n+1} \in [0, 1)$ be the infection frequency, $x(t_{n+1}^0) = 0.1\mathcal{K}$ (here $0.1\mathcal{K}$ can be replaced by other numbers in $(0, \mathcal{K})$ as the results shown below are nearly the same) be the number of wild mosquitoes, then the

number of infected mosquitoes is

$$y(t^0_{n+1}) = \frac{Q_{n+1}x(t^0_{n+1})}{1 - Q_{n+1}}. \tag{10}$$

We apply for a less laborious release with $R = 3$ and a higher female contamination rate $c = 1.5\%$. Figure 2C plots the dependence of S_{n+1} and P_{n+1} on Q_{n+1}. It is clearly seen that the two curves define a threshold value $Q_{n+1} \approx 20.5\%$, below which S_{n+1} stays constantly about 74.42% and P_{n+1} remains at approximately 5.05%. When $Q_{n+1} > 20.5\%$, S_{n+1} has a slightly decrease to reach 57.37% while P_{n+1} has a sudden jump to reach 100%. These interesting phenomena show that both S_{n+1} and P_{n+1} are nearly independent of Q_{n+1} for $Q_{n+1} < 20.5\%$, and $Q_{n+1} > 20.5\%$. As long as Q_{n+1} is maintained below the threshold value 20.5%, the mosquito population can be suppressed successfully with an annual suppression efficiency 74.42% and a moderate infection frequency 5.05%. Our calculations predict a sustainable mosquito control strategy with no risk of population replacement in consecutive years. By releasing HC mosquitoes with a constant ratio $R = 3$ and a contamination rate $c \leq 1.5\%$, about 75 percent (74.42%) of wild mosquitoes will be eliminated, and the annual infection rate at the end of each hot season will be maintained below 5% (5.05%).

Dependence of the maximal tolerance of HC female contamination rates on different suppression efficiencies. Before examining the dependence of the maximal tolerance of HC female contamination rates on different suppression efficiencies, we numerically seek the approximate release ratios to achieve population suppression by solving equations (3) and (4) with the parameter values and the initial conditions specified in

Table 1. Let $R(S_i)$ be the release ratio that produces a given suppression efficiency S_i. Then

$$R(50\%) \approx 1, \quad R(60\%) \approx 1.5, \quad R(80\%) \approx 3.92. \tag{11}$$

For instance, $R(50\%) \approx 1$ predicts that half of the mosquito population will be suppressed if released males have about an equal number of wild males.

We fix the R values at the three ratios given in (11) and simulate the curves of S_1 and P_1 against the female contamination rate c that increases from 0.5% to 50%. For clarity, the range of c in Figure 2F with $R = 3.92$ is from 0.5% to 5%. The upper limit 50% for c represents a reared mosquito population that is not sexually sorted at all, with an equal number of males and females. Such unsorted mosquito populations have been routinely used in the practice of population replacement. When the release ratio $R \leq 1$, Figure 2D shows that the mosquito population will not be completely replaced even if released mosquitoes are not sexually sorted, and the maximal infection rate is about 43.95%. The curve S_1 decreases in c linearly, while P_1 increases in c almost linearly. It was expected that the contamination of infected females could improve the suppression efficiency since these females gave birth to infected offspring, and increase the CI occurrence probability. However, this phenomenon has not been observed when $R \leq 1$ because the amount of accidentally released infected females and their offspring is not large enough to drag down the wild female mosquito population size.

However, if we increase the release ratio to 1.5 to pursue 60% suppression of wild mosquitoes, Figure 2E tells us that the maximal tolerance of HC female contamination

is about 18%. Different from Figure 2D, the largest release ratio in Figure 2E makes S_1 not monotonic for $c \in [0.5\%, 18\%]$: it undergoes a slight reduction from 60% to 49.86% when c goes from 0.5% to 16%, and then increases to 50.91% when c hits the maximal tolerance rate 18%, implying that appropriate amount release of infected females could improve the suppression efficiency.

Figure 2F shows that pursuing 80% suppression of wild mosquitoes drags the maximal tolerance of HC female contamination down to 1.45%. When $R = 3.92$ and $c = 1.45\%$, we have $S_1 \approx 79.32\%$ and $P_1 \approx 9.02\%$. In contrast, when $R = 1.5$ and $c = 1.78\%$, one has $S_1 \approx 59.10\%$ and $P_1 \approx 2.68\%$. This observation implies that for large release ratios, if there is no risk of population replacement, then the more accidentally released infected females, the higher the suppression efficiency. However, for the contamination rate resulting in $P_1 = 1$, for example, when $c = 20\%$, the suppression efficiency is about 57.38% for both $R = 1.5$ and $R = 3.92$ since wild females have been eradicated and infected females have been stabilized at their carrying capacity, whose magnitude is mainly determined by the fitness cost of infected females compared to wild females.

Supplemental References

[1] Zheng, B., Yu, J., Xi, Z., et al. (2018). The annual abundance of dengue and Zika vector *Aedes albopictus* and its stubbornness to suppression. Ecological Modelling 387: 38–48.

[2] Yan, Z., Hu, Z., Jiang, Y., et al. (2010). Factors affecting the larva density index of *Aedes albopictus* in Guangzhou. Journal of Tropical Medicine 10(5): 606–608.

[3] Liu, F., Yao, C., Lin, P., et al. (1992). Studies on life table of the natural population of *Aedes albopictus*. Acta Scientiarum Naturalium Universitatis Sunyatseni 31: 84–93.

[4] Vinogradova, E. B. (2007). Diapause in aquatic insects, with emphasis on mosquitoes. In Diapause in aquatic invertebrates theory and human use. V.R. Alekseev, B.T. Stasio, J.J. Gilbert, eds. (Springer Netherlands), 83-113.

[5] Zheng, B., Tang, M., Yu, J. (2014). Modeling *Wolbachia* spread in mosquitoes through delay differential equations. SIAM Journal on Applied Mathematics 74: 743-770.

Table S1. Experimental design for the wild female mating preference assay, indicating the allocation of different mosquito lines to each experimental group.

Treatment 1<br>szGUA♀: szGUA♂: HC♂	No. of szGUA♀	No. of szGUA♂	No. of HC	Total No. in the cage	Replicate
1:1:1	120	120	120	360	3
1:1:5	50	50	250	350	3
1:1:10	30	30	300	360	3
Treatment 2<br>xhGUA♀: szGUA♂: HC♂					
1:1:1	120	120	120	360	3
1:1:5	50	50	250	350	3
1:1:10	30	30	300	360	3
Control					
szGUA♀: szGUA♂	50	50	–	100	1
szGUA♀: HC♂	–	50	50	100	1

The *A. albopictus* lines labeled as szGUA and xhGUA represent wild *A. albopictus* obtained from Shazai Island or Xiaohu Island, respectively. The first-generation offspring of the field-collected mosquitoes were used in the experiment.

Table S2. Experimental results from the female mating preference assay.

R	Female origin	Replicate 1				Replicate 2				Replicate 3			
		Y	N	FMCI-1	FMCI-2	Y	N	FMCI-1	FMCI-2	Y	N	FMCI-1	FMCI-2
1:1	Shazai	34	51	0.60	0.40	35	44	0.56	0.44	33	52	0.61	0.39
	Xiaohu	36	41	0.53	0.47	41	42	0.51	0.49	31	30	0.49	0.51
1:5	Shazai	4	28	0.58	0.42	6	19	0.39	0.61	4	39	0.66	0.34
	Xiaohu	5	21	0.46	0.54	5	24	0.49	0.51	6	19	0.39	0.61
1:10	Shazai	1	10	0.50	0.50	1	19	0.66	0.34	1	13	0.57	0.43
	Xiaohu	2	15	0.43	0.57	1	17	0.63	0.37	1	16	0.62	0.38

R: ratio of GUA males to HC males.

Y: The count of GUA female individuals with viable eggs, indicating mating between GUA females and GUA males.

N: The count of GUA female individuals with unviable eggs, indicating mating between GUA females and HC males.

FMCI: female mating choice indices.

FMCI-1: FMCI to HC♂$= (N/R)/(Y + N/R)$.

FMCI-2: FMCI to GUA♂$= Y/(Y + N/R)$.

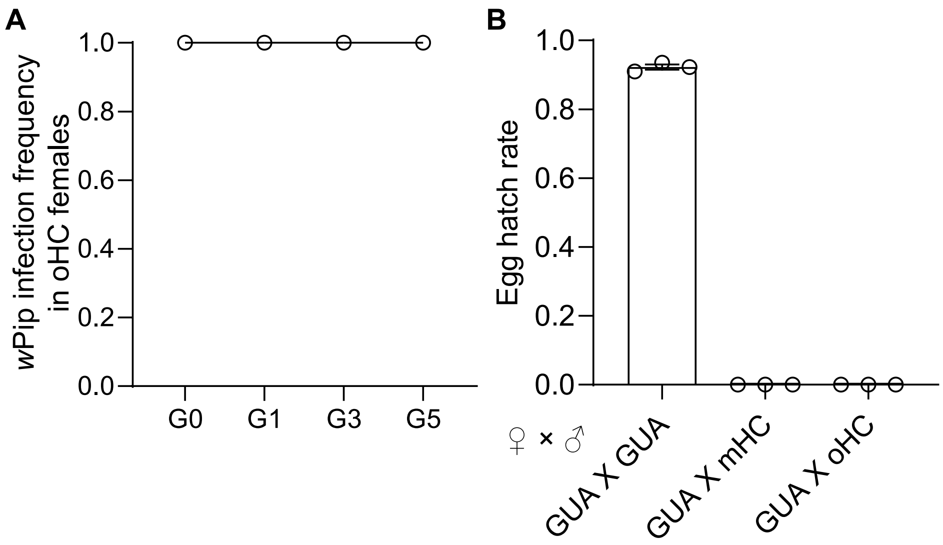

S1.pdf

Figure S1: The wPip infection rate and the CI induction ability of the oHC mosquito line. (A) The wPip infection frequency was monitored from G0 to G5, all females checked were wPip positive ($n = 8$ in each generation). (B) oHC induced 100% CI penetrance. Mean ± SEM, $n = 3$.

S2.pdf

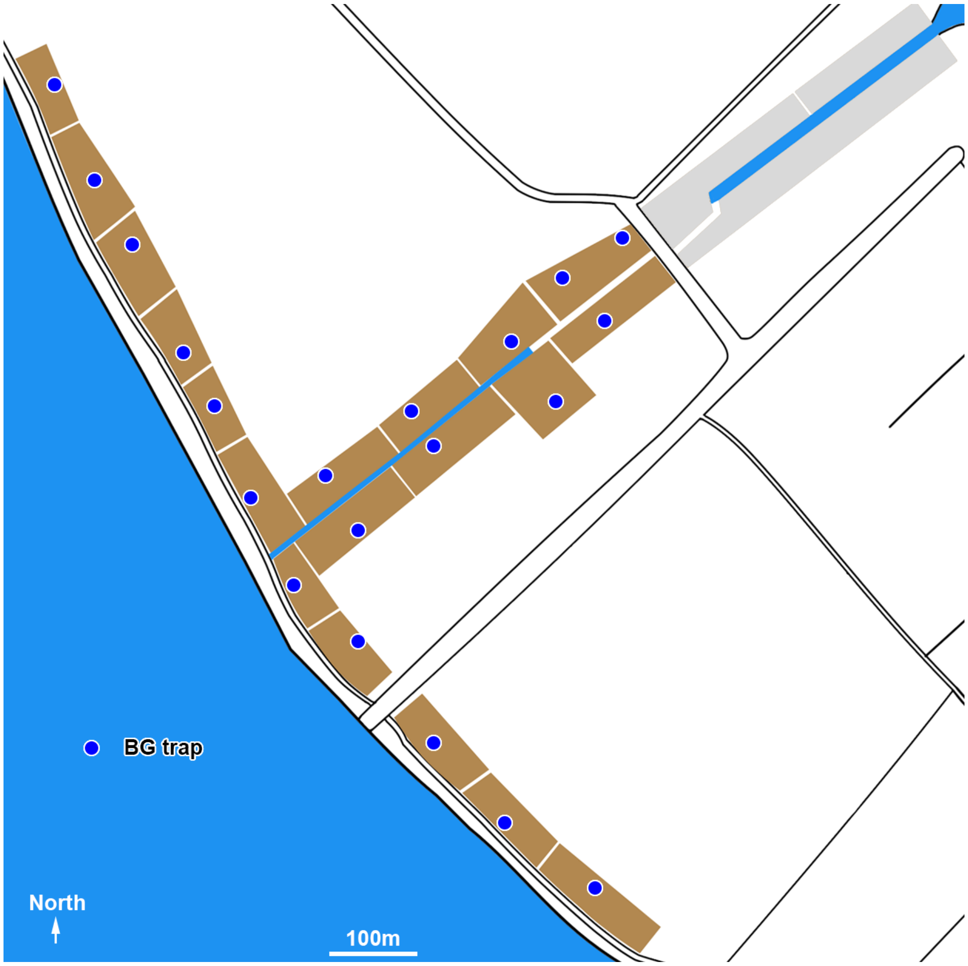

Figure S2. Illustration of BG trap distribution on Shazai Islands in 2019. There were 20 BG traps distributed on Shazai Island.

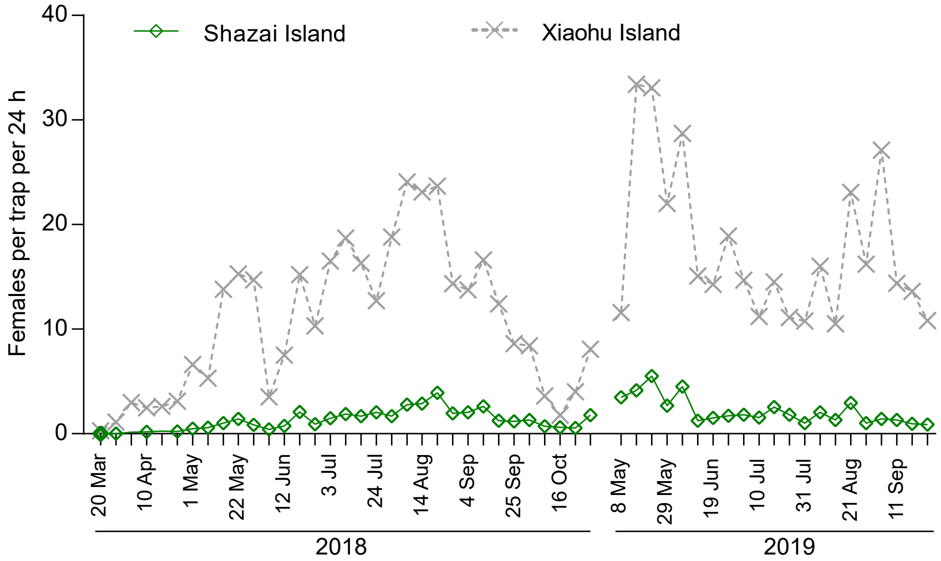

S3.pdf

Figure S3. The population size of *A. albopictus* adults on Shazai and Xiaohu islands when suppression was maintained by the standalone IIT. Data were presented as female mosquitoes per trap per 24 hours. Two-tailed Mann Whitney U test, $P < 0.0001$; $n = 33$ in 2018, and $n = 21$ in 2019.

S4.pdf

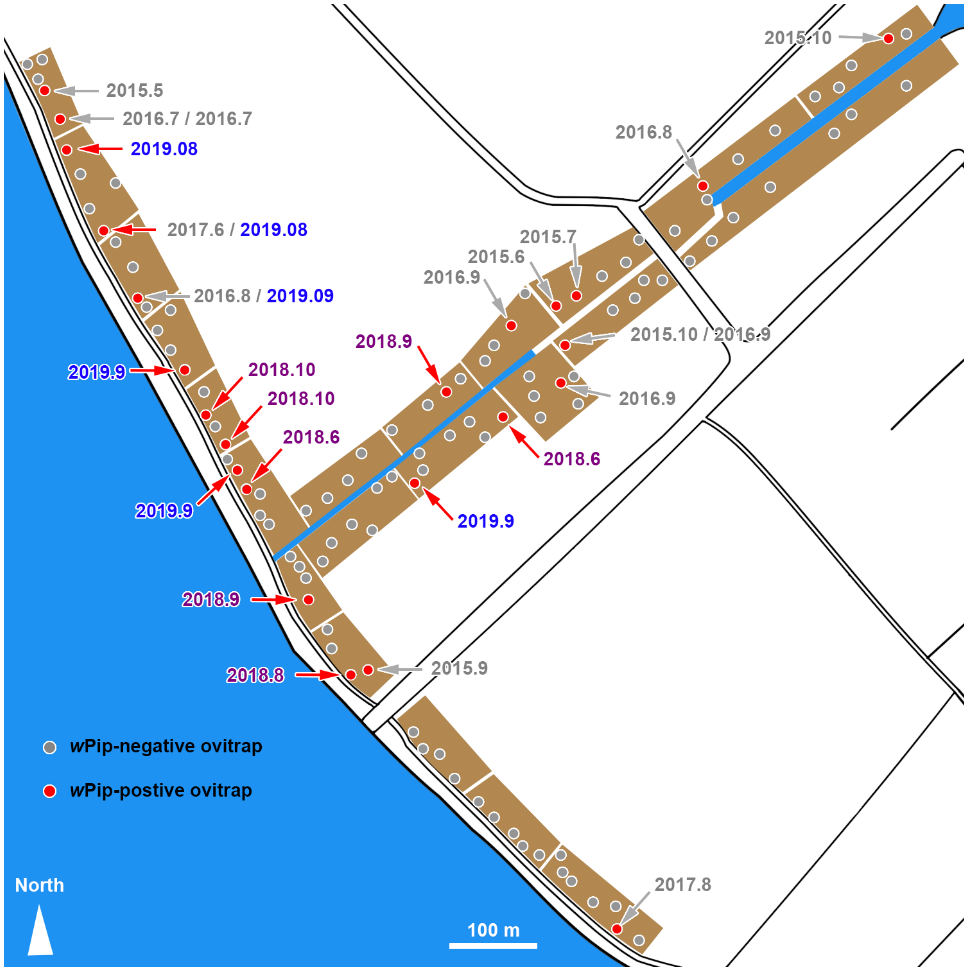

Figure S4. Temporal and spatial distribution of *w*Pip-positive ovitraps on Shazai Island across the entire mosquito population suppression years. The locations of the ovitraps are marked with circles on the map, with red circles indicating those in which *w*Pip-positive larvae were detected. Specifically in 2018 and 2019, *w*-positive ovitraps were identified, and their corresponding time points are color-coded in purple and blue, respectively.

S5.pdf

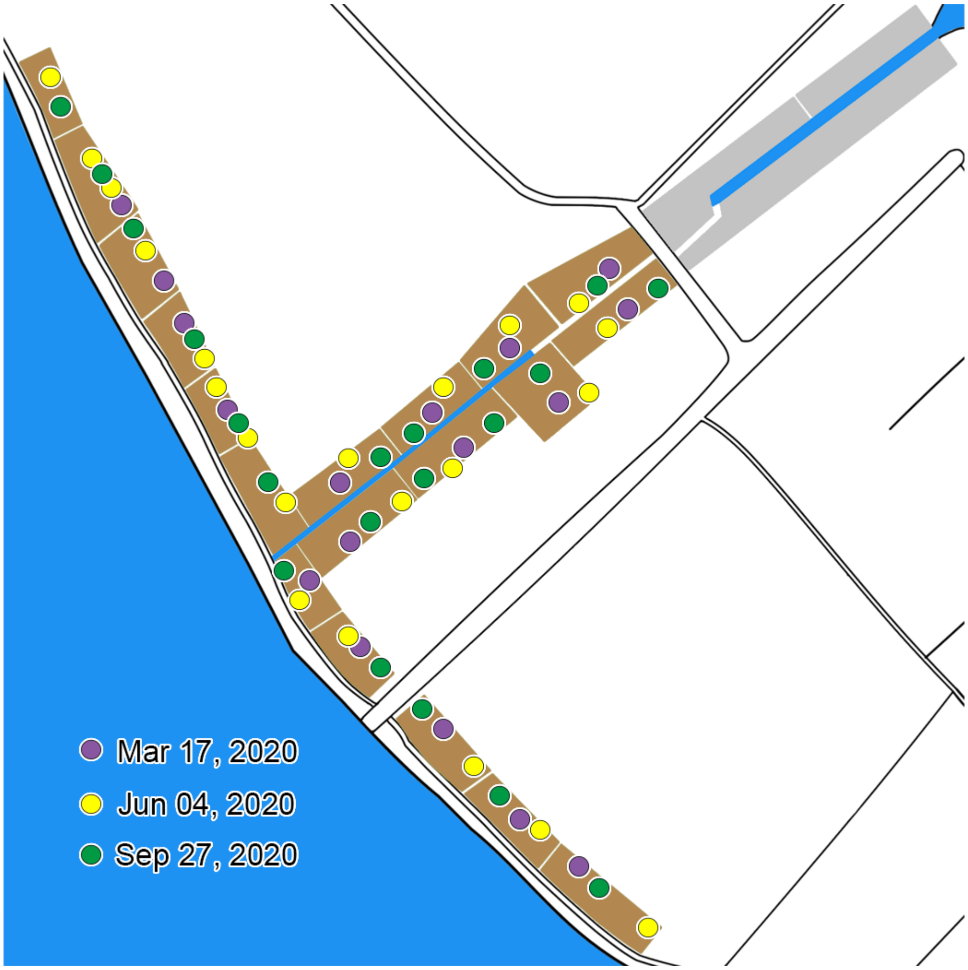

Figure S5. Map of locations where human landing catches were performed on Shazai Island in 2020. In 3 independent human landing catch activities, a total of 205 female mosquitoes in 58 distinct localities were collected.

S6.pdf

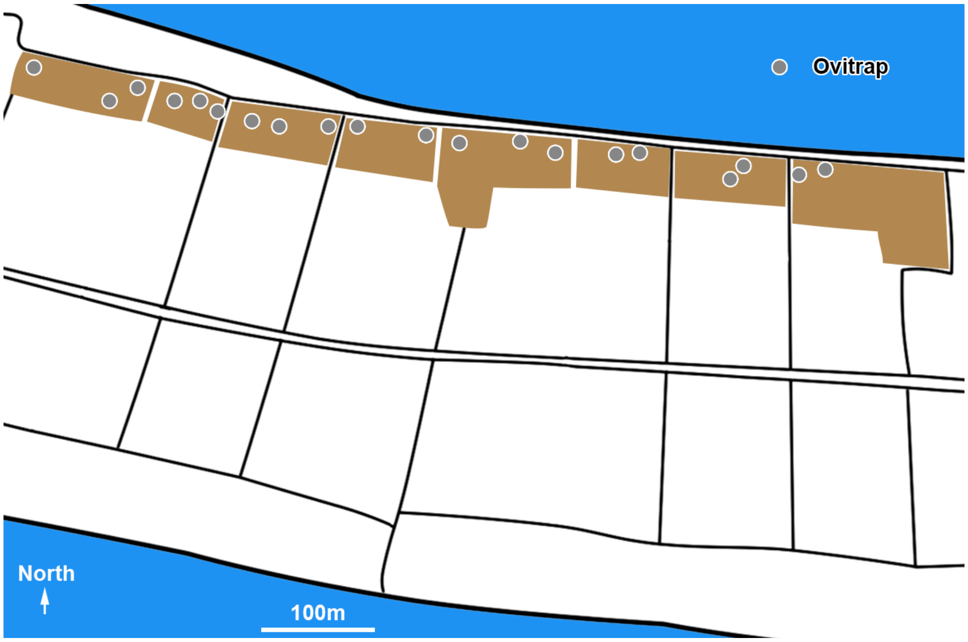

175 **Figure S6. Illustration of ovitrap distribution on the Dadaosha Islands in**
176 **2019.** There were 20 ovitraps distributed on Dadaosha Island.
